# Abnormal Anatomical Rich-Club Organization and Structural–Functional Coupling in Mild Cognitive Impairment and Alzheimer's Disease

**DOI:** 10.3389/fneur.2020.00053

**Published:** 2020-02-05

**Authors:** Rui Cao, Xin Wang, Yuan Gao, Ting Li, Hui Zhang, Waqar Hussain, Yunyan Xie, Jing Wang, Bin Wang, Jie Xiang

**Affiliations:** ^1^College of Software, Taiyuan University of Technology, Taiyuan, China; ^2^College of Information and Computer, Taiyuan University of Technology, Taiyuan, China; ^3^School of Life Science, Beijing Institute of Technology, Beijing, China; ^4^Department of Radiology, First Hospital of Shanxi Medical University, Taiyuan, China; ^5^Department of Neurology, Xuanwu Hospital, Capital Medical University, Beijing, China; ^6^Department of Health management, Aerospace Center Hospital, Peking University Aerospace School of Clinical Medicine, Beijing, China

**Keywords:** Alzheimer's disease, mild cognitive impairment, rich-club organization, SC-FC coupling, brain network

## Abstract

Emerging research indicates interruptions in the wiring organization of the brain network in Mild cognitive impairment (MCI) and Alzheimer's disease (AD). Due to the important role of rich-club organization in distinguishing abnormalities of AD patients and the close relationship between structural connectivity (SC) and functional connectivity (FC), our study examined whether changes in SC-FC coupling and the relationship with abnormal rich-club organizations during the development of diseases may contribute to the pathophysiology of AD. Structural diffusion-tensor imaging (DTI) and resting-state functional magnetic resonance imaging (fMRI) were performed in 38 normal controls (NCs), 40 MCI patients and 19 AD patients. Measures of the rich-club structure and its role in global structural–functional coupling were administered. Our study found decreased levels of feeder and local connectivity in MCI and AD patients, which were the main contributing factors to the lower efficiency of the brain structural network. Another important finding was that we have more accurately characterized the changing pattern of functional brain dynamics. The enhanced coupling between SC and FC in MCI and AD patients might be due to disruptions in optimal structural organization. More interestingly, we also found increases in the SC-FC coupling for feeder and local connections in MCI and AD patients. SC-FC coupling also showed significant differences between MCI and AD patients, mainly between the abnormal feeder connections. The connection density and coupling strength were significantly correlated with clinical metrics in patients. The present findings enhanced our understanding of the neurophysiologic mechanisms associated with MCI and AD.

## Introduction

The human brain is a complex network that supports efficient communication through structurally and functionally interconnected brain units (1). The motivation for studying the brain's underlying connectivity is the theory that the function of the brain depends on the network organization of the whole brain rather than individual nodes or individual connections (2–4). Generally, the brain connectome has been directly probed by structural connectivity (SC) derived from diffusion-tensor imaging (DTI), which represents anatomical wiring diagrams, and functional connectivity (FC) derived from resting-state functional magnetic resonance imaging (fMRI), which reflects the synchronization of neuronal activities in different brain regions (5, 6). Recently, researchers have argued that the functional network connections of the human brain are limited by the potential anatomical white matter pathway (5, 7–17). The association between SC and FC, called structural–functional (SC-FC) coupling, and the joint study of SC and FC can describe the functional dynamics of the brain from a structural topological perspective and may detect subtle brain changes more sensitively than any single imaging modality (7, 18, 19). A large number of studies have demonstrated that the abnormal functional dynamics in brain network dysfunction in schizophrenia, idiopathic generalized epilepsy, migraine, and Alzheimer's disease were caused by abnormalities due to brain diseases (1, 19–21).

Alzheimer's disease (AD) is a neurodegenerative disease that is clinically characterized by progressive memory impairment and loss of cognitive functions and is considered a syndrome of disconnectivity among brain regions (22). Mild cognitive impairment (MCI) is a prodromal stage of AD, with a conversion rate of 10–15% to AD annually (23). Studies using neuroimaging techniques, such as DTI and fMRI, suggested the destruction of white matter pathways or SC among the pathological regions attributed to abnormalities in FC and cognitive decline in AD patients (24). A large number of studies with AD patients showed SC and/or FC abnormalities among related brain regions, such as the hippocampus and precuneus (14, 25, 26). Dai et al. (21) demonstrated overlapping and distinct network disruptions in SC and FC in AD. By analyzing the functional dynamics of the brain from a structural topological perspective, Su et al. (11) observed a decrease in the strength of SC-FC coupling and speculated that decreased coupling may be suggestive of less dynamic and more stringent brain function in AD patients. Wang et al. (27) argued that the disruption of optimal structural organization may have given rise to alterations in functional dynamics. Recently, Dai et al. (21) reported that the increased functional dynamics of the default-mode network in AD patients indicated that AD leads to a strengthened relationship between FC and the underlying anatomical connectivity. More importantly, the disruptions in structural–functional relationships in patients with AD might be the primary cause of the cognitive deficits (28).

Graph theory analyses have shown changes in the topological properties of brain networks in AD, including decreases in global and local efficiency and impairments in small-world properties (21, 26, 29, 30). Rich-club organization is characterized by a tendency for hub regions to be more densely connected among themselves than with peripheral regions (31). As high-capacity centrical cores, the rich-club connections play a central part in the global neural information communication of the brain (32, 33). Previous network studies have reported overall level interruptions in the brain network of SC and FC in patients, together with significant lesions distributed throughout the hub regions in the frontal, temporal, and parietal cortices (34–36). A study by Mallio et al. (37) reported a trend of epicentral disruption around the entorhinal and hippocampal regions in MCI and AD patients, which is consistent with the transneuronal spread hypothesis. Daianu et al. (36) suggested that structural network disruptions predominated in more remotely connected regions in patients. In our previous studies, the disturbances in rich-club organization dynamically and potently disrupted connectivities among peripheral regions in preclinical AD and MCI patients, and the disrupted connectivities spread to the rich-club regions in the brains of patients with AD (26, 38). Compared with the rich-club connections, the feeder and local connections are demonstrated to be impaired earlier and more severely (36). Recently, Dai et al. (21) found that the rich-club connections had significantly increased SC-FC coupling in AD. But only AD patients were studied, the progressive changes at the early prodromal stage of neurodegenerative diseases were still unclear. Wang et al. (27) found that moderate CIND had higher SC-FC correlation than HC, while Sun et al. (11) observed a decrease in the strength of SC-FC coupling in AD patients. However, the results of the previous work are inconsistent, and the relationship between abnormal anatomical rich-club organization and disruptions in structural–functional relationships in MCI and AD patients remains unclear.

In this study, we examined the underlying abnormal anatomical rich-club organization using DTI and resting-state fMRI data from a group of 38 normal controls (NCs), 40 MCI patients and 19 AD patients. We anticipated that the functional dynamics of patients would change due to these disproportionately disrupted white matter connections during the progression of AD. Finally, the clinical relevance of the structural rich-club organization and SC-FC coupling was also examined in patients to promote a mechanistic understanding of the dynamic changes in clinical manifestations and identify a potential biomarker that can detect subtle brain connectivity disruption with high sensitivity.

## Materials and Methods

### Participants

A total of 97 participants were collected from the Alzheimer's Disease Neuroimaging Initiative (ADNI) database (http://adni.loni.ucla.edu), including 19 patients with AD (8 females), 40 patients with MCI (20 females), and 38 normal control subjects (19 females) (Table 1). The ADNI was approved by the ethics committee of the National Institute on Aging, the National Institute of Biomedical Imaging and Bioengineering, and the local ethics committee of each participating site. The participants also provided written informed consent when they registered for imaging and completed the questionnaires. All participants were assessed by a standardized clinical evaluation protocol, including a neurologic examination, a medical history interview, and a battery of neuropsychological tests (26). All participants were right-handed and had no history of neurological or psychiatric disorders. The neuropsychological tests included the Clinical Dementia Rating Scale (CDR) (39), Geriatric Depression Scale (GDS) (40), Functional Assessment Questionnaire (FAQ) (41), Mini-Mental State Examination (MMSE) (42), and Montreal Cognitive Assessment (MoCA) (43). The NCs with CDR scores of 0 and MMSE scores of 24–30 were classified as non-demented, non-MCI, and non-depressed. The MCI patients had CDR scores of 0.5 and MMSE scores of 23–30. The AD patients met the National Institute of Neurological and Communicative Disorders and Stroke and the Alzheimer's Disease and Related Disorders Association (NINCDS/ADRDA) criteria for probable AD and had CDR scores of 0.5 or 1 and MMSE scores of 14–26.

**Table 1:** Demographic characteristics and neuropsychological test results.

**Characteristic**	**NC (*n* = 38)**	**MCI (*n* = 40)**	**AD (*n* = 19)**	**Test statistic**	***P*-value**
Age (y)	75.1 ± 1.6	75.3 ± 1.1	74.6 ± 2.3	*F* = 0.04	0.96
Sex (male/female)	19/19	20/20	11/8	*x*2 = 0.381	0.826
Education level (y)	17.0 ± 0.4	15.6 ± 0.5	15.4 ± 0.5	*F* = 3.55	0.033^a^^b^
CDR	0.03 ± 0.02	0.49 ± 0.07	1.00 ± 0.11	*F* = 54.59	<0.001^a^^b^^c^
FAQ	0.08 ± 0.06	3.10 ± 0.77	15.06 ± 2.04	*F* = 39.33	<0.001^a^^b^^c^
MMSE	29.24 ± 0.17	27.90 ± 0.29	21.53 ± 1.08	*F* = 73.98	<0.001^a^^b^^c^
MoCA	25.00 ± 0.39	20.05 ± 0.91	13.87 ± 1.35	*F* = 48.68	<0.001^a^^b^^c^
GDS	0.50 ± 0.12	1.83 ± 0.25	1.84 ± 0.32	*F* = 12.84	<0.001^a^^b^

*^A^Values are given as the mean ± SD*.

*^B^NC, normal control; MCI, amnestic mild cognitive impairment; AD, Alzheimer's disease; CDR, Clinical Dementia Rating Scale; FAQ, Functional Activities Questionnaire; MMSE, Mini-Mental State Examination; MoCA, Montreal Cognitive Assessment; GDS, Geriatric Depression Scale*.

a *NC group and MCI patients showed significant differences (P < 0.05)*.

b *NC group and AD patients showed significant differences (P < 0.05)*.

c *MCI patients and AD patients showed significant differences (P < 0.05)*.

### Imaging

Brain imaging was performed for all participants with a 3-T Siemens scanner according to the ADNI acquisition protocol. Structural DTI data, T1-weighted data and resting-state fMRI data were collected from all participants. During the data acquisition, headphones and cushions were used to minimize subject motion artifacts and scanner noise. The DTI data of each subject were collected three times using an echo planar imaging (EPI) sequence with the following parameters: 30 independent directions; slices = 31; repetition time (TR) = 12400.0 ms; echo time (TE) = 95.0 ms; field of view (FOV) = 2088.0 × 2088.0 mm^2^; flip angle=90.0 degrees; acquisition matrix = 1044 × 1044; and slice thickness=2.0 mm. The resting-state fMRI data included 197 functional volumes and were acquired with the following parameters: TE = 30.0 ms; TR = 3000.0 ms; slice thickness = 3.4 mm; flip angle = 90.0 degrees; and 48 slices. During the data acquisition, all subjects were requested to relax their minds, open their eyes, and move as little as possible. T1-weighted MR images were obtained by a 3D magnetization-prepared rapid gradient echo (MPRAGE) using the following parameters: 176 sagittal slices; TR = 2300.0 ms; slice thickness = 1.0 mm; flip angle=9.0 degrees; TE = 2.98 ms; FOV = 240 × 256 mm^2^; and acquisition matrix = 240 × 256. All DTI data, T1-weighted data and resting-state fMRI data were checked for quality assurance to exclude scans with excessive motion and/or artifacts after preprocessing corrections; all scans were included.

### Image Preprocessing

Anatomical parcellation: Freesurfer software was used to segment the T1 images of each subject. Freesurfer uses a surface-based alignment procedure, which might be more accurate than a volume-based alignment of a cortical atlas (44). Freesurfer applies a standardized processing pipeline to the T1 image, including skull stripping, volumetric labeling, intensity normalization, white matter segmentation, surface atlas registration, surface extraction, and gyral labeling. We used the Brainnetome atlas (BN atlas) and provided parcellation of 210 cortical and 36 subcortical subregions (45). The cortical and subcortical parcellation of each individual in native T1 space was transformed to the native DWI or fMRI space by applying a rigid body transformation computed with the FMRIB Software Library (FSL, http://fsl.fmrib.ox.ac.uk/fsl/fslwiki) FLIRT tool (46).

#### DTI Preprocessing

We used the Pipeline for Analyzing braiN Diffusion imAges toolbox (PANDA, http://www.nitrc.org/projects/panda) to preprocess DTI data following a method illustrated previously (47). PANDA is a MATLAB toolbox for pipeline processing of diffusion MRI images based on the Diffusion Toolkit (http://www.trackvis.org/dtk/) and the FSL tool. First, we corrected for head motion and eddy current distortions. Subjects with more than 3 mm or degrees of head movement in any direction were removed. Then, we calculated the fractional anisotropy (FA) of each voxel and used the affine transformation to coregister FA image with the corresponding T1 image in the native space. After that, each region of the subject was defined as a brain network node. Reconstruction of WM pathways (referred to as fibers or tracts) for each individual DTI data using deterministic white matter tractography based on the fiber assignment by continuous tracking (FACT) algorithm (48, 49). Eight seeds followed the main diffusion direction from voxel to voxel in the brain mask. A streamline will terminate when the streamline reaches a voxel with an FA value <0.1, when the streamline exceeds the cerebral mask, or when the trajectory of the streamline makes a turn sharper than 45 degrees (1, 50).

#### fMRI Preprocessing

We used a toolbox for Data Processing & Analysis for Brain Imaging (DPABI) to preprocess resting-state fMRI data following a method illustrated previously (51). The first 10 volumes were discarded due to the instability of the initial signals before starting the preprocessing. Then, slice timing correction and head motion correction were performed for each subject (52–55). According to the criteria of spatial movement, subjects with more than 3 mm or degrees of head movement in any direction were removed. Next, the T1 image was registered to the mean head motion-corrected fMRI image. The functional images were then resampled to 3-mm isotropic voxels and spatially smoothed with a 4-mm full-width half-maximum (FWHM) Gaussian kernel. We regressed several nuisance signals, such as global mean signals, head motion signals (Friston's 24-parameter model), and signals from the cerebrospinal fluid and white matter from the data. To reduce the effects of high-frequency noise and low-frequency drift, we used linear detrending and bandpass filtering (0.01–0.1 Hz) (53). Finally, we extracted the mean time series from the 246 ROIs for each subject (56).

### Network Construction

**Figure 1:**
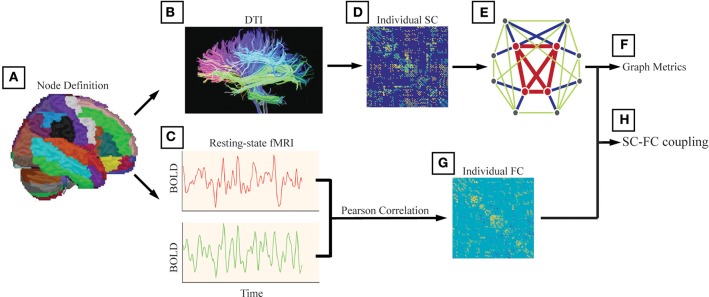
The analysis steps were performed following Li et al. (20). **(A)** We used the Brainnetome atlas (BN atlas) and provided parcellation of 210 cortical and 36 subcortical subregions. We also used the automated anatomical labeling (AAL) atlas and this parcellation divided the cortical surface into 90 regions (45 per hemisphere). The method and results are shown in Supplementary. **(B)** Structural connectivity (SC) was defined as at least 3 bundles of fibers (>2) between any two regions. **(C)** Functional connectivity (FC) between any two nodes was computed as the Pearson correlation between the blood-oxygenlevel dependent (BOLD) time series. **(D)** The weighted networks were calculated as the number of fibers divided by the sum of the surface areas of the 2 interconnected ROIs. **(E)** We generated a binary and undirected network for each participant with the structural connectivity between two regions set as 1 if the corresponding weight was positive (weight > 0). **(F)** The investigated graph metrics included the rich-club coefficient, the degree, the global efficiency, the local efficiency, and the nodal efficiency. **(G)** For each participant, we obtained a 246 × 246 symmetric FC matrix with Pearson's correlation coefficients as the weights, and negative correlation coefficients were set as zero. **(H)** The alterations in SC-FC coupling in AD patients and MCI patients were investigated.

Structural connectivity (SC) was defined as at least 3 bundles of fibers (>2) between any two regions (Figures 1A–E). In addition to analyzing unweighted networks, the weighted networks were calculated as the number of fibers divided by the sum of the surface areas of the 2 interconnected ROIs. Therefore, for each subject, we obtained a 246 × 246 weighted SC matrix.

Functional connectivity (FC) between any two nodes was computed as the Pearson correlation between the blood-oxygen-level dependent (BOLD) time series (Figures 1C–G). For each participant, we obtained a 246 × 246 symmetric FC matrix with Pearson's correlation coefficients as the weights, and negative correlation coefficients were set as zero due to the ambiguous biological explanation (57, 58).

### Structural Network Analysis

To investigate potential differences in the topology of the structural network between the patient groups and the NC group, characteristic graph metrics were calculated (binary and undirected 246 × 246 networks). We generated a binary and undirected network for each participant with the structural connectivity between two regions set as 1 if the corresponding weight was positive (weight > 0). Graph metrics are introduced in detail elsewhere (50, 59). The investigated metrics included the rich-club coefficient (defined as the ratio of connections present between the remaining nodes and the total number of possible connections that would be present if the set was fully connected) (1), the degree (computed as the sum of the node's connections), the global efficiency (reflecting the ability for network-wide communication), the local efficiency (reflecting how much the network is fault tolerant and shows how efficient the communication is among the first neighbors of the node i when it is removed), and the nodal efficiency (defined as the inverse of the harmonic mean of the minimum path length between an index node, i, and all the other nodes in the network) (60). All graph metrics and null models were computed using the MATLAB-based GRETNA toolbox (http://www.nitrc.org/projects/gretna/) (61). The results of the rich-club organization and nodal efficiency were visualized by the BrainNet Viewer toolbox (http://www.nitrc.org/projects/bnv) (62).

### Rich-Club Organization of Structural Network

**Figure 2:**
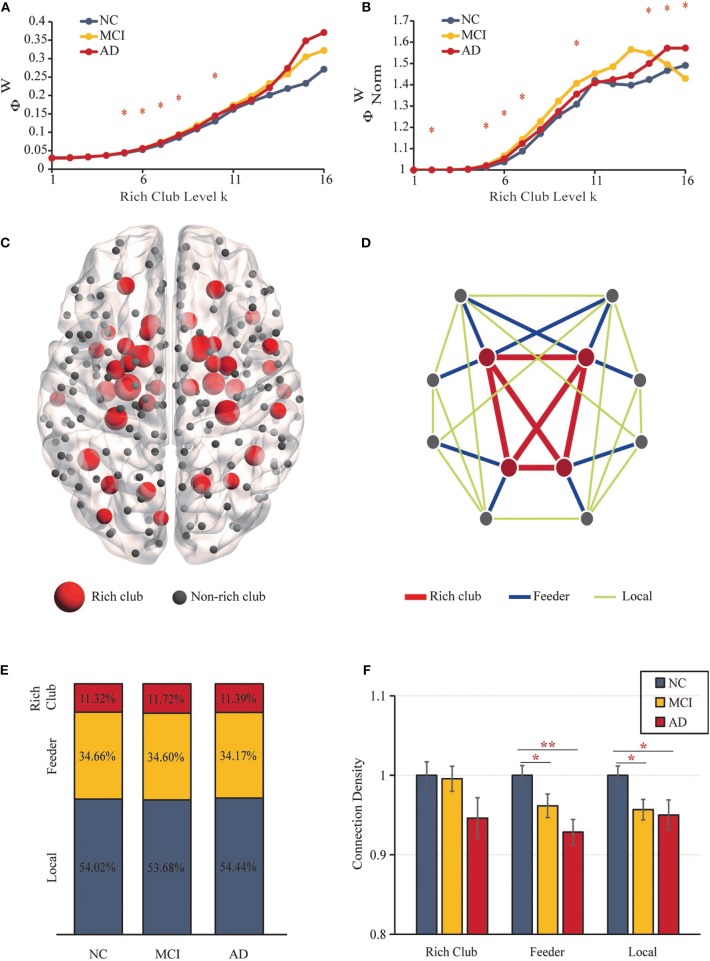
Rich-Club Organization. **(A)** The rich-club coefficients. **(B)** The normalized rich-club coefficients. **(C)** The rich-club nodes (red nodes) are shown across all groups. **(D)** The different kinds of connections in structural networks. **(E)** The proportions of three kinds of connections for each group. **(F)** Bar graphs display the mean (SD) density of the rich-club, feeder and local connections (**P < 0.01, *P < 0.05).

The “rich club” refers to nodes with higher degrees within brain networks and a higher connectivity strength of internodal connections compared to those composed of randomly selected brain regions (32). Rich-club nodes were chosen according to the top 18% of average node degree ranked nodes across all participants (26). According to the classification of rich-club and non–rich-club nodes, structural network connections were categorized into three types of edges (32): (i) rich-club connections (red), connecting rich-club regions to each other; (ii) feeder connections (blue), connecting rich-club regions to non–rich-club regions; and (iii) local connections (green) connecting non–rich-club regions to each other (Figure 2D).

### SC-FC Relationship Analysis

To measure the correlation between SC and FC and examine the alterations in SC-FC coupling in AD patients and MCI patients, we analyzed the correlation between the structural connection strength and functional connection strength of each network. This correlation was restricted by the connections with nonzero SC and nonzero FC. All nonzero entries of the SC matrix were selected, rescaled to a Gaussian distribution, and correlated with their nonzero functional counterparts selected from the FC matrix (1, 7, 11, 19–21, 27, 63–65). Therefore, for each of the subjects, we obtained a single SC-FC coupling metric.

### Statistical Analysis

All statistical analyses in this study were performed by the Statistical Package for Social Science (SPSS, v20.0) (http://www.spss.com/). We used ANOVA to test for group differences in age and education and used a chi-square test to test for differences in sex. ANCOVAs (corrected for age, sex, and education) were used to test for group differences in rich-club coefficients, normalized rich-club coefficients, three classes of connectivity density, other network topology metrics, nodal efficiency and SC-FC coupling, with Bonferroni corrections for multiple comparisons at *P* < 0.05. We used partial Pearson's correlations controlling for sex, age, and education to measure how SC-FC coupling, graph metrics and clinical performance related to the density of connections in each group. Significance was set at *P* < 0.05.

## Results

### Anatomical Rich-Club Organization

The structural brain network construction process is illustrated in Figure 1D. Figures 2A,B show rich-club coefficient curves *R(k)* and *R*_*norm*_*(k)* for NC (blue), MCI (yellow) and AD (red) groups. In our results, anatomical rich-club organization was obvious in three groups, with the normalized rich-club coefficient *R*_*norm*_*(k)* increasing as a function of node degree (*k*) higher than 1. In the whole-brain network, the rich-club coefficient *R(k)* showed a significant group difference (*k* = 5–8, 10; *P* < 0.05, ANCOVA; age, sex and education as covariates; Bonferroni corrected). Significant group differences in normalized rich-club coefficients *R*_*norm*_*(k)* for the ranges *k* = 2, 5–7, 10, and 14–16 reflect a higher level of connectivity between central hubs of the brain (*P* < 0.05, ANCOVA; age, sex, and education as covariates; Bonferroni corrected). Therefore, the rich-club organization of the structural brain networks in patients was significantly altered. The results for 90 brain regions are shown in Supplementary Figures S1A,B.

### Rich-Club Regions in SC

Rich-club regions were defined as the top 43 (*k* > 10) brain regions with the highest degree (Figure 2C, Table 2). Rich-club nodes were distributed in the Subcortical Nuclei (18 nodes, including Hippocampus, Basal Ganglia, and Thalamus), Temporal Lobe (10 nodes were located in Fusiform Gyrus, Superior Temporal Gyrus and Middle Temporal Gyrus), Frontal Lobe (6 nodes, including Orbital Gyrus, Precentral Gyrus and Inferior Frontal Gyrus), Parietal Lobe (4 nodes, including Precuneus and Inferior Parietal Lobule), Occipital Lobe (4 nodes were concentrated in the Medioventral Occipital Cortex), and Insular Lobe (1 node). This result is similar to the results of previous studies using the same atlas (66). The results for 90 brain regions are shown in Supplementary Figure S1C.

**Table 2:** List of rich-club nodes.

**Abbreviations**	**Gyrus**	**Lobe**	**Average node degree**	**MNI(X,Y,Z)**
Hipp_L_2_1	Hippocampus	Subcortical nuclei	19.351	−22, −14, −19
Hipp_R_2_2	Hippocampus	Subcortical nuclei	17.907	29, −27, −10
BG_R_6_5	Basal ganglia	Subcortical nuclei	16.835	14, 5, 14
Hipp_L_2_2	Hippocampus	Subcortical nuclei	16.464	−28, −30, −10
Hipp_R_2_1	Hippocampus	Subcortical nuclei	15.948	22, −12, −20
FuG_L_3_3	Fusiform gyrus	Temporal lobe	15.835	−42, −51, −17
BG_L_6_5	Basal ganglia	Subcortical nuclei	15.784	−14, 2, 16
FuG_L_3_1	Fusiform gyrus	Temporal lobe	14.557	−33, −16, −32
FuG_R_3_1	Fusiform gyrus	Temporal lobe	14.474	33, −15, −34
OrG_L_6_5	Orbital gyrus	Frontal lobe	14.309	−10, 18, −19
BG_R_6_3	Basal ganglia	Subcortical nuclei	14.134	15, 8, −9
FuG_R_3_3	Fusiform gyrus	Temporal lobe	14.134	43, −49, −19
BG_R_6_6	Basal ganglia	Subcortical nuclei	13.361	29, −3, 1
BG_L_6_6	Basal ganglia	Subcortical nuclei	13.34	−28, −5, 2
OrG_R_6_3	Orbital gyrus	Frontal lobe	12.784	23, 36, −18
FuG_L_3_2	Fusiform gyrus	Temporal lobe	12.639	−31, −64, −14
MVOcC_L_5_5	Medioventral occipital cortex	Occipital lobe	12.619	−13, −68, 12
MVOcC_R_5_5	Medioventral occipital cortex	Occipital lobe	12.598	15, −63, 12
PCun_R_4_3	Precuneus	Parietal lobe	12.289	16, −64, 25
OrG_R_6_5	Orbital gyrus	Frontal lobe	12.206	9, 20, −19
OrG_L_6_3	Orbital gyrus	Frontal lobe	12.103	−23, 38, −18
PCun_L_4_3	Precuneus	Parietal lobe	11.948	−12, −67, 25
BG_L_6_3	Basal ganglia	Subcortical nuclei	11.835	−17, 3, −9
Tha_L_8_7	Thalamus	Subcortical nuclei	11.753	−12, −22, 13
Tha_R_8_7	Thalamus	Subcortical nuclei	11.68	10, −14, 14
BG_L_6_4	Basal ganglia	Subcortical nuclei	11.619	−23, 7, −4
BG_R_6_2	Basal ganglia	Subcortical nuclei	11.247	22, −2, 3
FuG_R_3_2	Fusiform gyrus	Temporal lobe	11.165	31, −62, −14
BG_L_6_2	Basal ganglia	Subcortical nuclei	11.093	−22, −2, 4
IPL_R_6_6	Inferior parietal lobule	Parietal lobe	10.979	55, −26, 26
INS_L_6_6	Insular gyrus	Insular lobe	10.856	−38, 5, 5
BG_R_6_4	Basal ganglia	Subcortical nuclei	10.825	22, 8, −1
PrG_R_6_6	Precentral gyrus	Frontal lobe	10.784	51, 7, 30
BG_L_6_1	Basal ganglia	Subcortical nuclei	10.577	−12, 14, 0
STG_R_6_3	Superior temporal gyrus	Temporal lobe	10.577	51, −4, −1
IPL_L_6_1	Inferior parietal lobule	Parietal lobe	10.392	−34, −80, 29
Tha_L_8_4	Thalamus	Subcortical nuclei	10.361	−7, −14, 7
MTG_R_4_4	Middle temporal gyrus	Temporal lobe	10.351	58, −16, −10
MTG_L_4_4	Middle temporal gyrus	Temporal lobe	10.34	−58, −20, −9
MVOcC_R_5_4	Medioventral occipital cortex	Occipital lobe	10.32	18, −60, −7
IFG_R_6_5	Inferior frontal gyrus	Frontal lobe	10.278	42, 22, 3
STG_L_6_1	Superior temporal gyrus	Temporal lobe	10.144	−32, 14, −34
MVOcC_L_5_2	Medioventral occipital cortex	Occipital lobe	10.034	−5, −81, 10

### Density of Rich-Club, Feeder, and Local Connections

Figure 2D shows the different classifications of structural connections in brain networks. In the three groups, rich-club connections were found to include 11.32–11.72% of the total network density, the proportion of feeder connections ranged from 34.66 to 34.17%, and the percentage of local connections ranged from 53.68 to 54.44% (Figure 2E). No significant differences were detected between the three groups.

Figure 2F shows the mean (SD) density values for each of the 3 classes. The density of feeder (*F* = 5.435, *P* = 0.006) and local (*F* = 4.199, *P* = 0.018) connections revealed significant reductions (ANCOVA; age, sex, and education as covariates; Bonferroni corrected). No significant group differences were found in rich-club density (*F* = 1.941, *P* = 0.149), and no significant reductions in rich-club density were found in the AD vs. MCI (*P* = 0.851) or NC groups (*P* = 0.065) and MCI vs. NC (*P* = 0.088) groups. For feeder and local density, there were significant decreases in the MCI (feeder: *P* = 0.036; local: *P* = 0.014) and AD (feeder: *P* = 0.002; local: *P* = 0.021) groups relative to the NC group. However, there was no significant difference in feeder (*P* = 0.143) and local (*P* = 0.749) density in the AD vs. MCI groups (ANCOVA; age, sex, and education as covariates; Bonferroni corrected). The results for 90 brain regions are shown in Supplementary Figures S1D–F.

### Structural Network Graph Metrics

**Figure 3:**
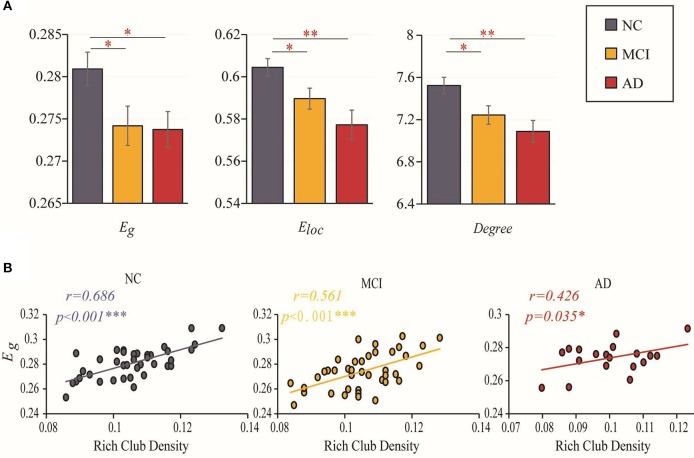
Efficiency and rich-club density. **(A)** Bar graphs display the mean (SD) global efficiency, local efficiency and degree. **(B)** The correlations between the global efficiency and the density of rich-club connections. The star-labeled numbers represent significant correlations (*P < 0.05, **P < 0.01, ***P < 0.001).

Disrupted topological organization of brain networks leads to altered information transmission efficiency. Group differences (ANCOVA; age, sex, and education as covariates; Bonferroni corrected) were observed in the global efficiency (*F* = 3.301, *P* = 0.041), local efficiency (*F* = 5.986, *P* = 0.004) and degree (*F* = 5.484, *P* = 0.006). Significant reductions in global efficiency (MCI vs. NC: *P* = 0.023; AD vs. NC: *P* = 0.050), local efficiency (MCI vs. NC: *P* = 0.027; AD vs. NC: *P* = 0.001) and degree (MCI vs. NC: *P* = 0.017; AD vs. NC: *P* = 0.003) were observed in the two patient groups compared to the NC group (ANCOVA; age, sex, and education as covariates). However, there was no significant difference between MCI and AD in these three topological properties (global efficiency: *P* = 0.904; local efficiency: *P* = 0.13; degree: *P* = 0.275) (Figure 3A). The results for 90 brain regions are shown in Supplementary Figure S2A.

### Relationship Between Global Efficiency and Rich-Club Density

We further investigated the relationship between the changes in rich-club organization and topologic alterations of the brain structural connectome. Figure 3B shows that the correlation of global efficiency to rich-club density decreased with worsening of disease status. A significantly positive correlation (with age, sex and education as covariates) was found between global efficiency and the rich-club density in the NCs (*r* = 0.686, *P* < 0.001), MCI patients (*r* = 0.561, *P* < 0.001) and AD patients (*r* = 0.426, *P* = 0.035). The results for 90 brain regions are shown in Supplementary Figure S2B.

### Altered SC-FC Coupling and Relationship to the Feeder and Local Density

Group differences (ANCOVA; age, sex, and education as covariates; Bonferroni corrected) were observed for the SC-FC coupling of all connections (*F* = 13.164, *P* < 0.001), feeder connections (*F* = 3.521, *P* = 0.034) and local connections (*F* = 8.325, *P* < 0.001). But the SC-FC coupling of rich-club connections (*F* = 1.863, *P* = 0.161) did not have group difference.

**Figure 4:**
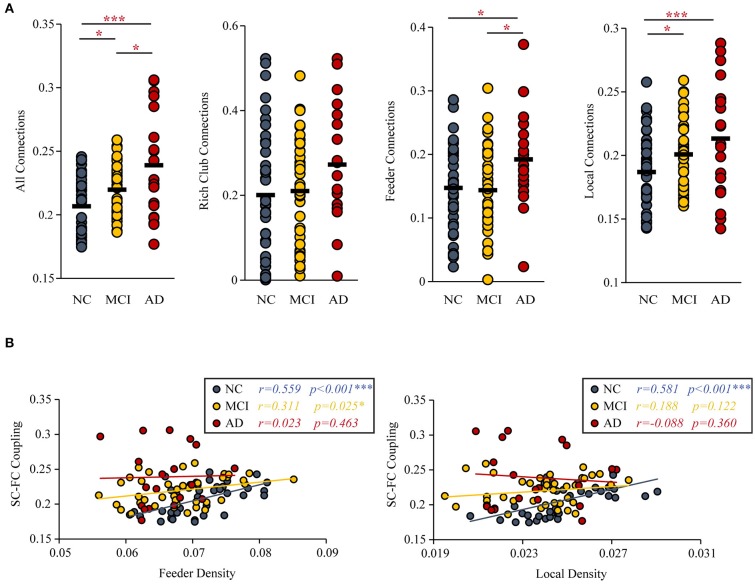
SC-FC Coupling. **(A)** MCI and AD patients had significantly increased SC-FC coupling compared with the NC group. **(B)** The correlations between the SC-FC coupling and the feeder and local density. The star-labeled numbers represent significant correlations (*P < 0.05, **P < 0.01, ***P < 0.001).

Under the constraint of existing structural connections, significant increases in the strength of SC-FC coupling were found (MCI vs. NC: *P* = 0.033; AD vs. NC: *P* < 0.001; AD vs. MCI: *P* = 0.011). Furthermore, the significantly increased SC-FC coupling was concentrated in feeder connections (AD vs. NC: *P* = 0.023; AD vs. MCI: *P* = 0.013) and local connections (AD vs. NC: *P* < 0.001; MCI vs. NC: *P* = 0.011) (Figure 4A).

The correlation (with age, sex, and education as covariates) of SC-FC coupling to feeder density (NC: *r* = 0.559, *P* < 0.001; MCI: *r* = 0.311, *P* = 0.025; AD: *r* = 0.023, *P* = 0.463) and local density (NC: *r* = 0.581, *P* < 0.001; MCI: *r* = 0.188, *P* = 0.122; AD: *r* = −0.088, *P* = 0.360) were decreased (Figure 4B). The results for 90 brain regions are shown in Supplementary Figure S3.

### Alzheimer's Disease-Related Alterations in Nodal Efficiency of SC

**Figure 5:**
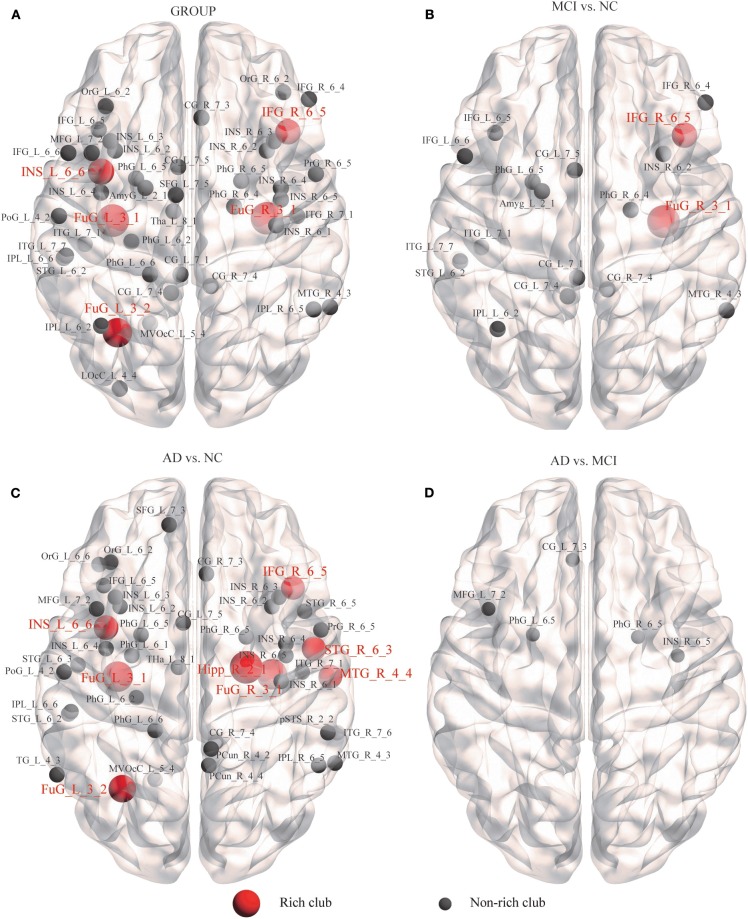
Alzheimer's disease-related alterations in regional efficiency and structural connectivity. We further examined the structural brain network nodes showing significant differences in nodal efficiency. **(A)** Group differences were observed for the 44 abnormal nodes. **(B)** MCI patients compared with the NCs. **(C)** AD patients compared with the NCs. **(D)** AD patients compared with MCI patients.

We further examined the structural brain network nodes showing significant differences in nodal efficiency (Figure 5), following the discovery of impaired rich-club organization. Group differences (ANCOVA; age, sex, and education as covariates; Bonferroni corrected) were observed for the 44 abnormal nodes, including 5 rich-club regions, IFG_R_6_5, the bilateral FuG_3_1, FuG_L_3_2, and INS_L_6_6, and 39 non–rich-club regions, which were located in the Temporal Lobe (10 nodes), Frontal Lobe (8 nodes), Insular Lobe (8 nodes), Limbic Lobe (5 nodes), Parietal Lobe (4 nodes), Occipital Lobe (2 nodes) and Subcortical Nuclei (1 node) (Figure 5A).

In the MCI group, 18 abnormal regions were found compared to the NC group, including 2 rich-club regions, IFG_R_6_5 and FuG_R_3_1, and 16 non–rich-club regions (Figure 5B). We found that the AD group had more abnormal nodes than the MCI group. When the AD patients were compared to the NCs, the 8 rich-club regions with altered nodal efficiency were Hipp_R_2_1, IFG_R_6_5, STG_R_6_3, MTG_R_4_4, bilateral FuG_3_1, FuG_L_3_2, and INS_L_6_6. The 38 non–rich-club regions were concentrated in the Temporal Lobe (14 nodes), Frontal Lobe (6 nodes), Parietal Lobe (5 nodes), Insular Lobe (8 nodes), Limbic Lobe (3 nodes), Subcortical Nuclei (1 node), and Occipital Lobe (1 node) (Figure 5C). Only 5 abnormal non–rich-club regions were found in AD patients compared with MCI patients (Figure 5D). The results for 90 brain regions are shown in Supplementary Figure S4.

### Relationship Among Connection Density, Coupling Metrics, and Clinical Performance

**Figure 6:**
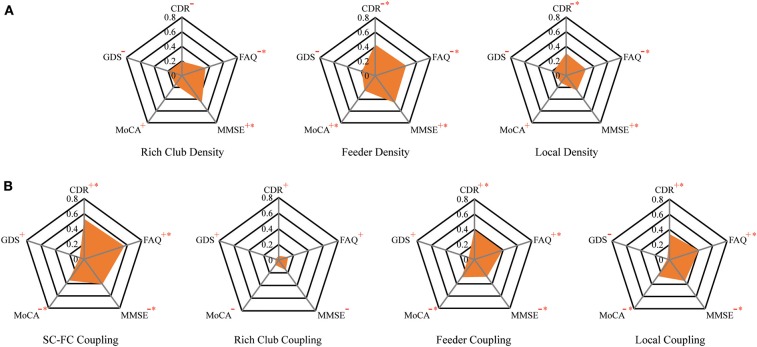
The relationship between connection density and coupling metrics and clinical performance. **(A)** The correlation between the clinical performance and the connection density. **(B)** The correlation between the clinical performance and the coupling metrics.

To investigate the relationship of the clinical and cognitive test variables and the altered brain networks in patients, we correlated the clinical and cognitive test variables with the anatomical rich-club organization and coupling strength (with age, sex, and education as covariates) (Figure 6, Table 3). In patients, the rich-club connection density was significantly correlated with FAQ and MMSE scores, the feeder connection density was significantly correlated with the CDR, FAQ, MMSE, and MoCA scores, and the local connection density was significantly correlated with the CDR, FAQ, and MMSE scores (Figure 6A). The coupling strength of all connections, feeder connections and local connections were significantly correlated with the CDR, FAQ, MMSE, and MoCA scores (Figure 6B). However, no significant correlations were found between the coupling strength of rich-club connections and any clinical variables. The results for 90 brain regions are shown in Supplementary Figure S5, Supplementary Table S1.

**Table 3:** Partial Pearson's correlations between connection density and coupling metric with clinical performance.

**COV: Sex & Age**		**CDR**	**FAQ**	**MMSE**	**MoCA**	**GDS**
Rich-club density	*r*	0.193	**0.339**	**0.461**	0.152	0.192
	*P*	0.099	0.011^*^	0.001^**^	0.157	0.101
Feeder density	*r*	**0.428**	**0.446**	**0.459**	**0.252**	0.193
	*P*	0.001^**^	0.001^**^	0.001^**^	0.046^*^	0.100
Local density	*r*	**0.304**	**0.284**	**0.254**	0.123	0.179
	*P*	0.020^*^	0.028^*^	0.044^*^	0.208	0.117
SC-FC coupling	*r*	**0.531**	**0.559**	**0.409**	**0.347**	0.062
	*P*	<0.001^***^	<0.001^***^	0.002^**^	0.009^**^	0.342
Rich-club coupling	*r*	0.054	0.126	0.155	0.075	0.042
	*P*	0.361	0.201	0.153	0.310	0.391
Feeder coupling	*r*	**0.392**	**0.389**	**0.283**	**0.291**	0.079
	*P*	0.004^**^	0.004^**^	0.028^*^	0.025^*^	0.302
Local coupling	*r*	**0.339**	**0.406**	**0.345**	**0.266**	0.031
	*P*	0.011^*^	0.003^**^	0.009^**^	0.037^*^	0.420

Partial Pearson's correlations controlled for age and sex were used to assess how connection density and coupling related to clinical performance. The star-labeled numbers represent significant correlations (^*^P < 0.05, ^**^P < 0.01,^***^P < 0.001). Bold Font indicated significant correlation (P < 0.05).

## Discussion

We investigated patterns of AD-related changes in brain structural networks and functional brain dynamics. Our main findings are as follows: (1) The feeder and local density were significantly decreased. Disrupted topological organization of structural networks leads to low information transmission efficiency. (2) Compared with NCs, MCI, and AD patients exhibited an increase in SC-FC coupling that was concentrated on the feeder and local connections. SC-FC coupling also displayed significant differences between MCI and AD patients that were mainly focused on abnormal feeder connections. (3) The alterations of regional efficiency in patients were observed, and most were peripheral regions. (4) The connection density and coupling strength were significantly correlated with clinical metrics in patients.

### Altered Anatomical Rich-Club Organization

An increasing normalized rich-club coefficient (*R*_*norm*_) > 1 over a range of degrees (k) reflects the existence of a rich-club organization in structural brain networks (1, 36, 50). Compared with NCs, the rich-club organization of the structural brain networks in patients was significantly altered, as indicated by the striking group difference in rich-club coefficients. To further confirm our findings, we selected 43 nodes with an average node degree >10 in the structural network across all participants as the hub node. Rich-club nodes mainly included the Hippocampus, Precuneus, and Fusiform Gyrus, which were consistent with previous studies (26, 67). In addition to the rich-club connection, the feeder and local connections showed more significantly damaged in MCI and AD patients (Figure 2F), which was consisted with previous studies. (26) found that the brain regions with the most aberrant connections involving peripheral and rich club regions in patients compared with NC were distributed throughout the whole brain. Moreover, most significant differences in nodal efficiency were observed in peripheral regions. AD is considered as a disease of extensive connectivity disorders, that peripheral regions and connections were the most vulnerable even in preclinical stages, the peripheral regions are more likely to suffer disruptions.

The node degree of MCI and AD patients was significantly reduced, indicating that the topological organization of the patient was damaged. Disrupted topological organization of brain networks affects the integration of information propagated among distant brain regions (68, 69). After the brain's long-distance signal traffic passed through a focal network, particularly in the central rich club, it depended on the feeder connections to facilitate or promote a variant of neuronal information for different parts of the network (32). We further investigated the relationship between the changes in anatomical rich-club organization and topologic alterations of the brain structural connectome and found that the brains of patients seemed to be less efficiently organized. The efficiency and degree of the MCI and AD patients were significantly decreased compared to those of the NC patients, and this finding was consistent with previous graph theory studies on AD (29, 30). We found that the decrease in structural network global efficiency was closely associated with the decrease in three types of connection density. As shown in Figure 2E, the proportion of the three connections in the three diagnostic groups remained generally stable. There were slight differences (but not significantly differences) of the rich-club density in the MCI and AD patients decreased relative to the NCs, so not all connections contributing to the highly interconnected nodes of the rich-club were kept intact during the stages of the disease. The failure of a rich-club region can have a severe effect on the level of global communication efficiency of a network due to its central role in the network (70, 71). More importantly, we found that with worsening of disease status from MCI to AD, the correlation between the global efficiency and the rich-club density was reduced. Corresponding to previous studies, the more central regions of the rich-club, which connect to remote nodes, may be relatively resistant to the neurodegenerative process (36). Peripheral regions were more susceptible due to their reduced persistence and lower level in the hierarchical network (26). Therefore, the rich-club connections are more stable than the feeder and local connections. As the condition worsened, the damage to the feeder and local connections intensified, and the impact on the global efficiency far exceeded the impact of the abnormal rich-club connections.

These findings indicated that the main cause of the lower global efficiency of the patient was the abnormal disruption of the overall structural connectivity. However, the rich-club density did not decrease significantly in the patients, suggesting that the most important factors were the densities of the feeder and local connections.

### The Relationship Between the Increase in SC-FC Coupling and Rich-Club Organization

We observed an increased level of coupling between SC and FC in the MCI and AD patients, which was consistent with previous studies (21, 27). Moreover, Dai et al. reported that the increased SC-FC coupling of the default-mode network in AD patients indicated that AD leads to a strengthened relationship between FC and the underlying anatomical connectivity (21, 27). However, one previous study reported the opposite result: a decrease in the strength of SC-FC coupling in AD patients (11). We found that the method of network construction in this study was different from the other two studies, and we speculated that these differences might lead to inconsistent findings. The increased coupling means a loss of reorganization of the brain network in AD and MCI (72), which likely reflects a worsening of disease status. Specifically, the degree of SC integrity might reflect the capacity of the cerebral cortex to maintain functional organization diversity or neural activity interactions (27, 73). Therefore, the increased coupling may suggest that the disease leads to functional interactions that are more directly related to the underlying anatomical connectivity of the brain and may be indicative of more stringent and less dynamic brain function and less functional network reorganization in patients (1, 21).

More importantly, further analysis revealed that the increased SC-FC coupling in the MCI and AD patients was concentrated on the feeder and local connections, which indicated that SC-FC coupling may detect subtle brain connectivity disruption with greater sensitivity than does a single modality (21, 27). Specifically, the change in the correlation between the structural network and the functional network of the NCs and MCI patients was mainly focused on abnormal local connections. The feeder connections of the structural network of MCI patients were also damaged, but there was no significant change in the SC-FC coupling of the feeder connections. We speculate that this observation was due to the compensation mechanism of the brain; patients also present adaptive network reorganization associated with relatively preserved cognitive function (74). However, these mechanisms are transient and attenuated (72). Compared with MCI, feeder connections are the main reason for the increase in SC-FC coupling in AD patients. As disease status worsens from MCI to AD, the disturbance of the feeder and local connections of the structural network become more serious, and the corresponding functional connections are greatly affected (7, 75). Therefore, the increase in SC-FC coupling in AD patients was mainly concentrated on feeder and local connections compared to that in NCs. Our study of changes in SC-FC coupling may contribute to a more accurate understanding of the dynamic changes in brain network structure in patients.

With worsening of disease status from MCI to AD, the coupling strengths were decreasingly correlated with density in the feeder and local connection, indicating a lower density of feeder and local connections accompanied by altered functional brain dynamics. Consistent with previous studies, destruction of feeder and local connections would contribute to the disruption of an optimal structural organization in AD and MCI patients (36). That an increased level of SC-FC coupling may be related to the destruction of the optimal structure led to not only weaker neural communication (27) but also lower functional brain dynamics. The significant correlation between the feeder connections and the SC-FC coupling disappeared in the AD patients, and the significant correlation between the local connections and the SC-FC coupling disappeared during the MCI phase. These results confirmed the previous speculation that MCI patients' structure-feeder connection still maintains relatively stable brain functional dynamics due to the compensation mechanism of the brain. Alternatively, FC is more robust and resilient against pathological attacks than SC (76), and FC might be less vulnerable and may even serve as a compensation mechanism for reduced SC in the face of early cognitive decline (77).

### Alterations of Regional Efficiency of SC in Patients

Through the analysis of nodal efficiency, significant group differences were observed for a large number of abnormal nodes, and most were peripheral regions, suggesting that AD is a disease of widespread connectivity disorders mainly based on feeder and local connections, which corresponded to our previous results. The efficiency of nodes such as the inferior frontal gyrus, amygdala, and temporal lobe (parahippocampal gyrus, superior temporal gyrus, parahippocampal gyrus) in the MCI patients was abnormally altered, which reflected the abnormality of the connections in these regions. These regions are involved in high-level cognitive functions, such as episodic memory, attention, motivation, self-awareness, and audiovisual integration, which are the main deficiencies in patients (e.g., frontal lobe, temporal lobe and insula). Previous studies have demonstrated AD-related abnormalities in the frontal lobe (35, 78), temporal lobe (79–81), and insula (35, 82). Moreover, with worsening of disease status from MCI to AD, the involvement of more nodes, and more severe damage to the structural brain network, the AD patients have more abnormal nodes than the MCI patients, including the precuneus, hippocampus and middle temporal gyrus, which are related to memory and cognitive function. The precuneus is involved in not only the rich-club nodes of the brain organization in this study but also the central nodes of the default-mode network in previous studies, and these regions have been implicated in high-level cognitive functions, including episodic memory, self-related processing, and aspects of consciousness (83). The frontal regions (e.g., middle frontal gyrus (MFG)) are thought to be involved in emotion, memory, and executive functions (84, 85). Many previous studies have demonstrated that these frontal regions exhibited AD-related abnormalities in structural network integrity (86, 87), gray matter morphology (88), and functional interactions (89–91).

### Association of Structural Connection Density With Coupling and Clinical Metrics

Abnormal connection densities were correlated with all the clinical and cognitive test variables in patients. The high correlations between the structural connection densities and the behavioral scores indicated that the connection densities were highly associated with the disrupted cognitive/memory functions (78). Consistent with previous studies, these findings converged on the notion that all AD-related patients present widespread aberrant connections involving the peripheral regions, which may contribute to the early decline in memory that they experience (26). The number of clinical scales significantly associated with feeder and local density was more than that associated with rich-club density, which was consistent with our findings. We hypothesized that feeder and local connections are more susceptible to damage in the course of the disease and thus have a greater impact on cognitive performance, while rich-club connections are more robust. The stable rich-club connections help to maintain the core organization of the brain when other rich-club regions become disrupted (50). Human SC is closely related to FC (92) and underlies high-order cognitive activities (93). Therefore, our results of disrupted SC may reflect the interruption of functional connections that affects the cognitive performance of the patients. The rich-club regions of the brain play a core role in optimizing global brain communication and are associated with higher cognitive functions (21). Abnormal rich-club region can have a serious effect on the brain's higher cognitive function due to its central role in the network. Although there was no significant difference in the rich-club density in our study, there was still a downward trend. Therefore, we found that rich-club density was significantly correlated with the FAQ and MMSE scores, and these results did not contradict our conclusions.

To our knowledge, this is the first study demonstrating a close association between greater SC-FC coupling and neuropsychological tests, including the CDR, FAQ, MMSE, and MoCA in patients. The results indicated that with the development of the disease, the level of the correlation between the structure and function of the brain increases, brain function becomes more stringent and less dynamic, and less functional network reorganization occurs in patients. Moreover, the clinical manifestations of patients were more obvious. Similarly, we observed that the coupling strength of the feeder and local connections were also significantly correlated with the four neuropsychological tests. However, the SC-FC coupling of the rich-club connections was not significantly correlated with any scale score. This finding is consistent with our results and further demonstrates that our results are reasonable and credible. SC-FC coupling may provide a potential biomarker that is more sensitive than a single modality to uncover the pathophysiology of AD (1, 19).

### Limitations and Future Work

Although we provided information on additional network properties and functional network reorganization in patients with MCI and AD, there were some limitations in the present study. The number of AD patients is quite small compared to the number of MCI patients and healthy controls. Regarding the limitation of the dataset, we are unable to collect more suitable AD data for experimentation. Whether the organization of SC and FC in patients have selective disruptions that disproportionately involve module-related or node-related connections remains to be determined. In our study, we calculated the correlation between SC and FC as SC-FC coupling, which was limited by the connections with non-zero SC and FC. As reported by Honey et al. (7) strong functional connections commonly exist between regions with no direct structural connection. Furthermore, due to the limitations of the method, there is currently no way to calculate the relationship between the functional connections and the indirect structural connections. The goal of these studies was simply to focus on the relationship between functional connections and structural connections. We will add the case of the indirect connections to the calculation of the SC-FC coupling in the next study. Besides, we have not studied whether the use of higher thresholds for functional connections will affect the results of SC-FC coupling. We plan to conduct such research in the future to improve the robustness of the results. Moreover, due to the cross-sectional design we used, the causal relationship between the abnormal rich-club organization and accompanying changes in brain dynamics remains unclear.

## Conclusion

In conclusion, our study found decreased levels of feeder and local connectivity in MCI and AD patients, which were the main contributing factors to the lower efficiency of the brain structural network. Disrupted topological organization of brain networks leads to low information transmission efficiency. The alterations of regional efficiency in patients further confirmed our findings. The MCI patients showed an intermediate position between the NC group and AD patients, and the AD patients had more abnormal nodes, including the rich-club nodes, than the MCI patients. Another important finding was that we have more accurately characterized the changing pattern of functional brain dynamics. The enhanced coupling between SC and FC in MCI and AD patients might be due to disruptions in optimal structural organization. More interestingly, we also found increases in the SC-FC coupling of feeder local connections in MCI and AD patients. SC-FC coupling also displayed significant differences between MCI and AD patients that were mainly focused on abnormal feeder connections. These findings suggest that robust rich-club and feeder connections help maintain stable brain functional dynamics. Finally, we examined whether the connection density and coupling strength were significantly correlated with clinical metrics in patients. In summary, our findings suggest that AD may break the hierarchical structure of the brain network, leading to functional network reorganization and communication network abnormally. SC-FC coupling may provide a potential biomarker that is more sensitive than a single modality to discover the pathophysiology of AD. The present findings enhanced our understanding of the neurophysiologic mechanisms associated with MCI and AD from a brain network perspective.

## Data Availability Statement

Publicly available datasets were analyzed in this study. This data can be found here: http://adni.loni.usc.edu/.

## Ethics Statement

The studies involving human participants were reviewed and approved by The Alzheimer's Disease Neuroimaging Initiative (ADNI) database. The patients/participants provided their written informed consent to participate in this study.

## Author Contributions

BW, RC, JX, and XW designed the study. RC, XW, YG, and JX did the experiments and data acquisition. XW, YG, and HZ performed analysis and interpretation of data. XW, YG, TL, and WH wrote the draft of the manuscript. TL, YX, and JW contributed to revise. All authors approve the manuscript to be published and agreed on all aspects of the work.

### Conflict of Interest

The authors declare that the research was conducted in the absence of any commercial or financial relationships that could be construed as a potential conflict of interest.
