# Supplementary material for: Abnormal Anatomical Rich-Club Organization and Structural–Functional Coupling in Mild Cognitive Impairment and Alzheimer's Disease
Source: Front Neurol. 2020 Feb 5;11:53. doi: 10.3389/fneur.2020.00053 (PMC7013042; doi:10.3389/fneur.2020.00053)
Supplement: Supplementary file 1 [file Data_Sheet_1.PDF]

Supplementary Material

Methods

This part of the method is only for the results in the supplemental material.

Image preprocessing

Image preprocessing steps were performed using the PANDA toolbox (http://www.nitrc.org/projects/panda) based on the FMRIB Software Library (FSL 5.0) for all DTI images (http://fsl.fmrib.ox.ac.uk/fsl/fslwiki) and the Diffusion Toolkit (http://www.trackvis.org/dtk/), including motion and eddy current corrections (Cui et al., 2013). The fractional anisotropy (FA) of each voxel was computed, with higher values indicating more directionally restricted diffusion of water molecules. Affine transformation was used to coregister FA images in native space to their corresponding T1-weighted images. Structural images were then nonlinearly registered to the ICBM152 template. Based on the above two steps, an inverse warping transformation from the standard space to the dMRI native space can be obtained. The automated anatomical labeling (AAL) atlas in the standard space was then used to inversely warp images back to individual native space by applying this inverse transformation. This parcellation divided the cortical surface into 90 regions (45 per hemisphere). The resulting inverse deformation map (T-1) for each subject was then applied to warp the AAL template to the DTI native space of each subject using the nearest neighbor interpolation method, as each AAL region was defined as a brain network node. Finally, for each individual DTI data set, WM pathways were reconstructed and referred to as fibers or tracts using streamline tractography. The fiber assignment by continuous tracking (FACT) algorithm was used (Mori and van Zijl, 2002; Mori et al., 2010). In the brain mask, 8 seeds followed the main diffusion direction from voxel to voxel. A streamline was terminated when it reached a voxel with an FA value lower than 0.1 (reflecting low levels of preferred diffusion, often gray matter voxels), when the streamline exceeded the brain mask (i.e., gray and white matter voxels), or when the trajectory of the streamline made a turn sharper than 45 degrees (Heuvel and Sporns, 2011; Mp et al., 2013).

The resting-state fMRI data preprocessing were coregistered with the T1 scan using Statistical Parametric Mapping (SPM8, http://www.fil.lon.ucl.ac.uk/software/SPM8/; Wellcome Trust Center for Neuroimaging, University College, London) and Data Processing Assistant for Resting-State fMRI (DPARSF) software (Yan and Zang, 2010) running under MATLAB 2012a. Briefly, prior to preprocessing, the first 10 volumes were discarded considering the instability of the initial signals and the subjects' adaptation to the environment. Then, the preprocessing procedures included slice timing correction and head-motion correction (Mowinckel et al., 2012; Power et al., 2012; Satterthwaite et al., 2012; Van Dijk et al., 2012). All data used in this study satisfied the criteria of

spatial movement in any direction < 3 mm or degrees. To spatially normalize the fMRI data for each individual, the T1-weighted image was first coregistered to the mean head-motion corrected fMRI image, segmented using DARTEL and transformed into Montreal Neurological Institute (MNI) space with the custom template generated from the group data. Notably, such a custom template could reduce the inaccuracy of spatial normalization due to the gray matter atrophy in MCI and AD patients. The parameters estimated in the DARTEL segmentation were applied to the fMRI data and thus normalized the fMRI data in MNI space. The functional data were further resampled to 3-mm isotropic voxels and spatially smoothed with a 4-mm full-width half-maximum (FWHM) Gaussian kernel. Then, several nuisance signals, including head-motion signals (Friston's 24-parameter model), global mean signals, and signals from the cerebrospinal fluid and white matter, were regressed from the data. Finally, linear detrending and bandpass filtering (0.01–0.1 Hz) were performed to reduce the effects of low-frequency drift and high-frequency noise (Power et al., 2012). Mean time series were subsequently extracted from the 90 AAL ROIs for each subject and paradigm to minimize the effects of the length of the time series on the derived reliability estimates of the graph properties (Braun et al., 2012).

Structural connectivity (SC) definition

A network consists of a set of nodes and connections that can be mathematically expressed as a graph: G = (V,E), with V indicating the collection of nodes and E the collection of edges between the nodes. A network was created: a whole brain network consisting of the 90 regions. The structural connection between nodes i and j was defined if a set of fibers (>0) exists between these two regions in the total collection of reconstructed fiber tracts (Figure 1A to E). In addition to analyzing unweighted networks, in which only information of the existence of a connection is present, an analysis was performed in which the SC was computed as the number of streamlines divided by the sum of the surface areas of the 2 interconnected ROIs.

Functional connectivity (FC) definition

The level of functional coupling (generally referred to as "functional connectivity" or FC) of a connection between 2 nodes i and j was computed as the Pearson cross-correlation between the resting-state fMRI time series of i and j (Figure 1C to G). To directly compare the structural and functional networks, the nodes of the functional network were analyzed in a similar way as the nodes in the structural network. Therefore, for each participant, we obtained a 90 × 90 symmetric FC matrix with Pearson's correlation coefficients as the weights.

Rich-club organization of the structural brain network

The edge architecture of brain networks can be characterized by using a rich-club

organization, which is defined as those connections among hub regions ("rich clubs"). The "rich club" refers to nodes with higher degrees within brain networks and a higher connectivity strength of internodal connections compared to those composed of randomly selected brain regions (Heuvel et al., 2012). To investigate the rich-club organization in patients, we adopted the approach described by van den Heuvel (2011, 2012) in the current study. In short, the degree of each node was first calculated, which is defined as the number of nodes across the brain showing a connection with the target node. All nodes that exhibited degree≤k were removed from the network. For the remaining network, the rich-club coefficient *R(k)* was defined as the ratio of connections present between the remaining nodes and the total number of possible connections that would be present if the set was fully connected (Heuvel and Sporns, 2011; Heuvel et al., 2012):

$$R(k) = \frac{2E_{>k}}{\boldsymbol{N}_{>k}(\boldsymbol{N}_{>k}-1)},$$

where k is the number of connections of node i, $N{>}k$ is the number of remaining nodes and $E_{>k}$ is the number of remaining connections that remove the nodes with connections less than k. $R_{random}(k)$ was computed as the average over a set of 1000 random graphs within the same number of nodes and nodal degree sequence. $R_{norm}(k)$ was computed as the ratio of $R(k)$ to $R_{random}(k)$:

$$R_{norm}(k) = \frac{R(k)}{R_{random}(k)}.$$

For a specified value of degree k, a network is considered to have a rich-club organization if $R_{norm}(k)>1$.

Rich-club nodes

Rich-club regions were selected on the individual level by ranking the nodes on the basis of a rich-club level for each data set and on the basis of the top 18% of most consistently ranked nodes across the group of subjects (Heuvel and Sporns, 2011).

Results

Rich-club organization

Figure S1A and S1B show rich-club coefficient curves R(k) and Rnorm(k) for NC (blue), MCI (yellow) and AD (red) groups. In our results, rich-club organization was evident in all groups, with the normalized rich-club coefficient Rnorm(k) increasing as a function of node degree (k) higher than 2. In the whole-brain network, rich-club coefficient R(k) showed a significant group difference (k = 6-9, 15-17; P < 0.05, Bonferroni corrected). Significant group differences in normalized rich-club coefficients Rnorm(k) for the ranges k = 13-14 and 17-18 reflect a higher level of

connectivity between central hubs of the brain ($P < 0.05$, Bonferroni corrected).

Rich-club regions

Selecting the rich-club regions on a group level was consistent with selecting rich-club regions on the individual level and across the group of subjects. Rich-club regions were defined as the top 16 ($k > 18$) brain regions with the highest degree. Rich-club nodes were as follows (in order of degree): right and left precuneus (bilateral PCUN), right lenticular nucleus, putamen (PUT.R), left lenticular nucleus, pallidum (PAL.L), right and left superior temporal gyrus (bilateral TPOsup), left calcarine fissure and surrounding cortex (CAL.L), left lingual gyrus (LING.L), right caudate nucleus (CAU.R), right hippocampus (HIP.R), right superior frontal gyrus, dorsolateral (SFGdor.R), left cuneus (CUN.L), right calcarine fissure and surrounding cortex (CAL.R), left middle occipital gyrus (MOG.L), right lingual gyrus (LING.R), and left hippocampus (HIP.L) (Figure S1C).

Density of rich-club, feeder, and local connections

Figure S1D shows the different classifications of structural connections in brain networks. In the three groups, rich-club connections were found to include 7.76%-8.08% of the total network density, the proportion of feeder connections ranged from 36.60% to 37.14%, and the proportion of local connections ranged from 54.79%-55.59% (Figure S1E). No significant differences were detected between the three groups.

Figure S1F shows the mean (SD) density values for each of the 3 classes. As the disease progressed, the density of feeder ($F = 4.530$, $P = 0.013$) and local ($F = 5.769$, $P = 0.004$) connections revealed significant reductions (ANOVA; Bonferroni corrected). Significant reductions in rich-club density were found in the AD versus NC groups ($P = 0.03$), but no significant difference was found in the AD versus MCI ($P = 0.596$) and MCI versus NC ($P = 0.491$) groups. For feeder and local density, there were significant decreases in the MCI (feeder: $P = 0.012$; local: $P = 0.003$) and AD (feeder: $P = 0.013$; local: $P = 0.012$) groups relative to the NC group. However, there was no significant difference in feeder ($P = 0.592$) and local ($P =0.887$) density in the AD versus MCI groups (ANCOVA; age, sex and education as covariates; Bonferroni corrected).

Network graph metrics

Group differences (ANOVA; Bonferroni corrected) were observed in the global efficiency ($F = 3.972$, $P = 0.022$) and degree ($F = 5.982$, $P = 0.004$) but not in local efficiency ($F = 1.019$, $P = 0.365$). Significant reductions in global efficiency (MCI versus NC: $P = 0.003$; AD versus NC: $P =0.007$) and degree (MCI versus NC: $P = 0.009$; AD versus NC: $P = 0.012$) were observed in the two patient groups compared to the NC group (ANCOVA; age, sex and education as covariates) (Figure S2A).

Relationship between global efficiency and rich-club density

Figure S2B shows that the correlation of global efficiency to rich-club density increased as the disease progressed. A significantly positive correlation (with age and sex as covariates) was found between global efficiency and the density of rich-club connections in the NCs ($r = 0.584$, $p < 0.001$), MCI patients ($r = 0.731$, $p < 0.001$) and AD patients ($r = 0.751$, $p < 0.001$). This finding indicated that the abnormally decreased global efficiency in AD had a close relationship with the lower level of connectivity of the rich-club connections.

Altered SC-FC coupling and relationship to the rich-club density

Group differences (ANOVA; Bonferroni corrected) were observed for all connections ($F = 4.617$, $P = 0.012$) and local connections ($F = 7.155$, $P = 0.001$). Under the constraint of existing structural connections, AD patients ($p = 0.007$) and MCI patients ($p = 0.021$) showed increases in the strength of SC-FC coupling compared with the NCs. Moreover, the significantly increased SC-FC coupling was concentrated in local connections (AD versus NC: $p = 0.002$; MCI versus NC: $p = 0.002$) (Figure S3A).

A moderate negative correlation (with age and sex as covariates) was found between feeder density and SC-FC coupling in the NC group ($r = -0.448$, $p = 0.006$), and this correlation was absent in the MCI group ($r = 0.108$, $p = 0.520$) and AD group ($r = -0.111$, $p = 0.673$) (Figure S3B).

Alzheimer’s disease-related alterations in regional efficiency

Following the discovery of a disrupted rich-club organization, we further identified the brain regions showing significant differences in regional efficiency (Figure S4). Group differences (ANOVA; Bonferroni corrected) were observed for the 27 abnormal nodes, including 7 rich-club regions, CAL.L, LING.R, bilateral PCUN, CAU.R, PUT.L, and TPOsup.R, and 20 non–rich-club regions, which were mainly located in the parietal lobe, temporal lobe and frontal lobe (Figure S4A).

In the MCI group, 14 abnormal regions were found compared to the NC group, including 2 rich-club regions, the bilateral PCUN, and 12 non–rich-club regions (Figure S4B). We found that the AD group had more abnormal nodes than the MCI group. When the AD patients were compared to the NCs, the 8 rich-club regions with altered nodal efficiency were CAL.L, CUN.L, bilateral LING, bilateral PCUN, CAU.R, and TPOsup.R. The 20 non–rich-club regions were concentrated in the frontal lobes, parietal lobes, temporal lobe and occipital lobe (Figure S4C). Compared to MCI patients, AD patients had 9 abnormal regions, including 4 rich-club regions, HIP.L, LING.R, PUT.L, and TPOsup.R, and 20 non–rich-club regions that were mainly distributed in the frontal lobe (Figure S4D).

Relationship among connection density, coupling metrics and clinical performance

To investigate the relationship of the clinical and cognitive test variables and the altered brain networks in patients, we correlated the clinical and cognitive test variables with the rich-club organization and coupling strength (with age and sex as covariates) (Figure S5, Table S1). In patients, the rich-club connection density was significantly correlated with CDR scores ($p < 0.05$, Table S1), the feeder connection density was significantly correlated with the MoCA and MMSE scores, and the local connection density was significantly correlated with the CDR, FAQ, MoCA and GDS scores (Figure S5A). The coupling strength of all connections and feeder connections was significantly correlated with the CDR and GDS scores (Figure S5B). The rich-club coupling was significantly correlated with the GDS scores. However, no significant correlations were found between the coupling strength of local connections and any clinical variables.

References

Braun, U., Plichta, M.M., Esslinger, C., Sauer, C., Haddad, L., Grimm, O., et al. (2012). Test–retest reliability of resting-state connectivity network characteristics using fMRI and graph theoretical measures. *NeuroImage* 59(2), 1404-1412. doi: http://dx.doi.org/10.1016/j.neuroimage.2011.08.044.

Cui, Z., Zhong, S., Xu, P., He, Y., and Gong, G.J.F.i.H.N. (2013). PANDA: a pipeline toolbox for analyzing brain diffusion images. 7(42), 42.

Heuvel, M.P., Van Den, Kahn, R.S., Joaquín, G.I., and Olaf, S.J.P.o.t.N.A.o.S.o.t.U.S.o.A. (2012). High-cost, high-capacity backbone for global brain communication. 109(28), 11372-11377.

Heuvel, M.P.V.D., and Sporns, O. (2011). Rich-Club Organization of the Human Connectome. *Journal of Neuroscience the Official Journal of the Society for Neuroscience* 31(44), 15775-15786.

Mori, S., ., Crain, B.J., Chacko, V.P., and Zijl, P.C., Van %J Annals of Neurology (2010). Three-dimensional tracking of axonal projections in the brain by magnetic resonance imaging. 45(2), 265-269.

Mori, S., and van Zijl, P.C.M. (2002). Fiber tracking: principles and strategies - a technical review. *NMR in biomedicine* 15(7-8), 468-480. doi: 10.1002/nbm.781.

Mowinckel, A.M., Thomas, E., and Westlye, L.T., %J Neuroimage (2012). Network-specific effects of age and in-scanner subject motion: a resting-state fMRI study of 238 healthy adults. 63(3), 1364-1373.

Mp, V.D.H., Sporns, O., Collin, G., Scheewe, T., Mandl, R.C., Cahn, W., et al. (2013). Abnormal rich club organization and functional brain dynamics in schizophrenia. *Jama Psychiatry* 70(8), 783-792.

Power, J.D., Barnes, K.A., Snyder, A.Z., Schlaggar, B.L., and Petersen, S.E., %J Neuroimage (2012). Spurious but systematic correlations in functional connectivity MRI networks arise from subject motion. 59(3), 2142-2154.

Satterthwaite, T.D., Wolf, D.H., Loughead, J., Ruparel, K., Elliott, M.A., Hakonarson, H., et al. (2012). Impact of in-scanner head motion on multiple measures of functional connectivity: Relevance for studies of neurodevelopment in youth ☆. 60(1), 623-632.

Van Dijk, K.R., Sabuncu, M.R., and Buckner, R.L.J.N. (2012). The influence of head motion on intrinsic functional connectivity MRI. 59(1), 431-438.

Yan, C.G., and Zang, Y.F.J.F.i.S.N. (2010). DPARSF: A MATLAB Toolbox for "Pipeline" Data Analysis of Resting-State fMRI. 4(13), 13.

Table

Table S1: Partial Pearson's correlations between connection density and coupling metric with clinical performance

COV: Gender & Age		CDR	FAQ	MMSE	MoCA	GDS
Rich Club Density	r	**0.230**	0.124	0.207	0.175	0.008
	P	**0.042***	0.179	0.061	0.096	0.476
Feeder Density	r	0.190	0.083	**0.224**	**0.224**	0.212
	p	0.078	0.270	**0.047***	**0.047***	0.057
Local Density	r	**0.240**	**0.227**	0.159	**0.263**	**0.281**
	P	**0.036***	**0.044***	0.118	**0.024***	**0.017***
SC-FC Coupling	r	**0.255**	0.081	0.014	0.097	**0.352**
	p	**0.028***	0.274	0.459	0.238	**0.004****
Rich Club Coupling	r	0.050	0.119	0.021	0.142	**0.257**
	P	0.356	0.188	0.437	0.147	**0.027***
Feeder Coupling	r	**0.292**	0.024	0.014	0.131	**0.343**
	p	**0.014***	0.429	0.460	0.165	**0.005****
Local Coupling	r	0.070	0.115	0.002	0.065	0.112
	P	0.303	0.197	0.494	0.315	0.203

Partial Pearson's correlations controlled for age and gender were used to assess how connection density and coupling related to clinical performance. The star-labeled numbers represent significant correlations (*P<0.05, ** P<0.01).

Figures

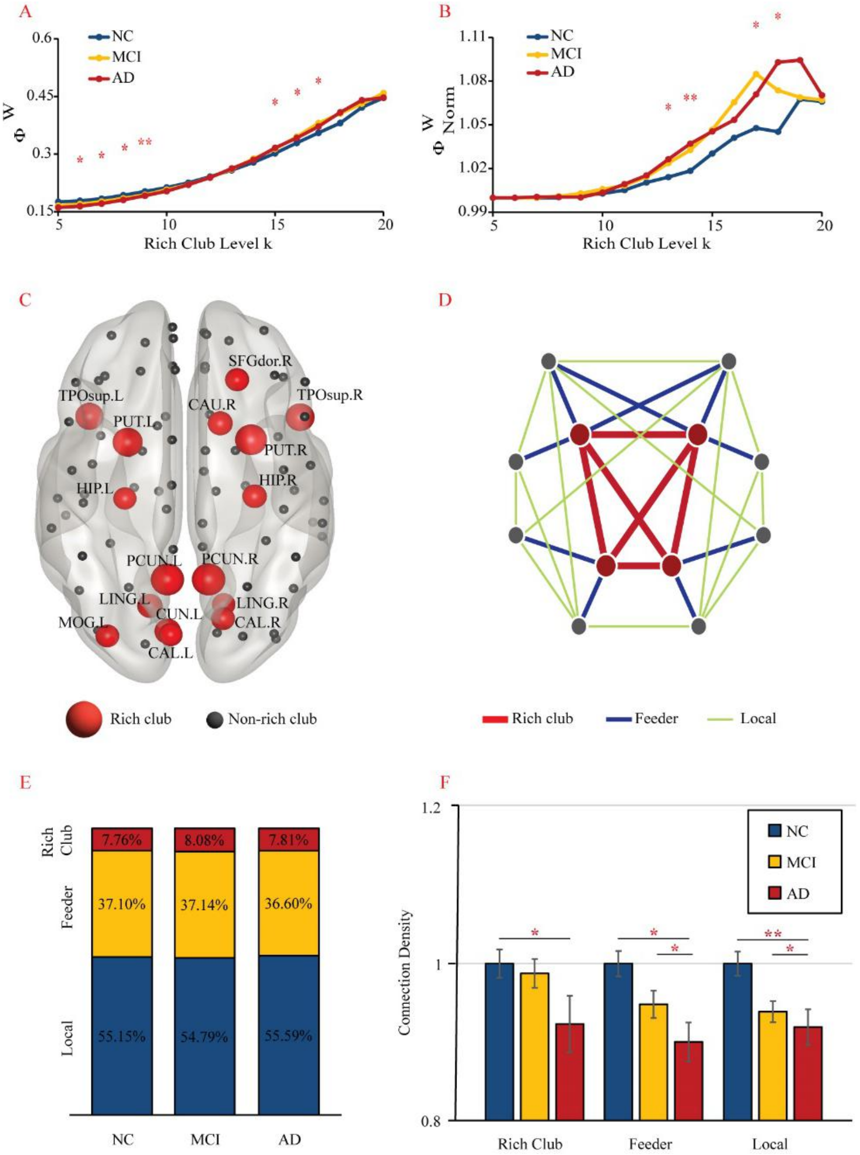

Figure S1. Rich-Club Organization. (A) The rich-club coefficients. (B) The normalized rich-club coefficients. (C) The rich-club nodes (red nodes) are shown across all groups. (D) The different kinds of connections in structural networks. (E) The proportions of three kinds of connections for each group. (F) Bar graphs display the mean (SD) density of the rich-club, feeder and local connections (** $P<0.01$, * $P<0.05$).

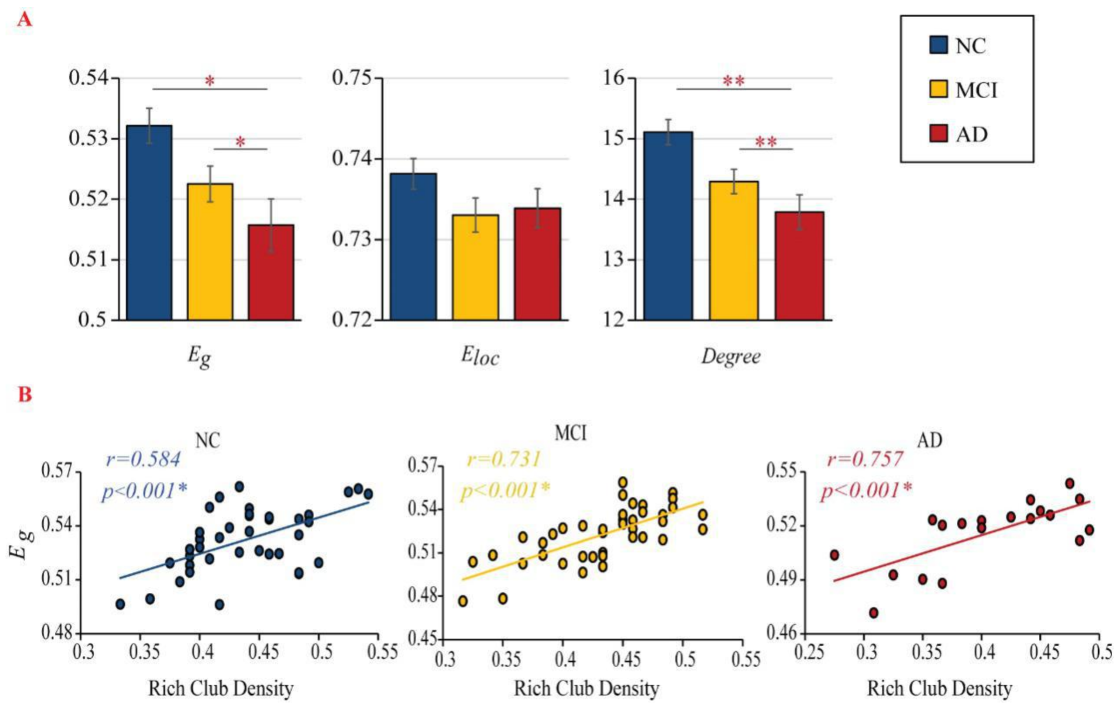

Figure S2. Efficiency and Rich-Club Density. (A) Bar graphs display the mean (SD) global efficiency, local efficiency and degree. (B) The correlations between the global efficiency and the density of rich-club connections.

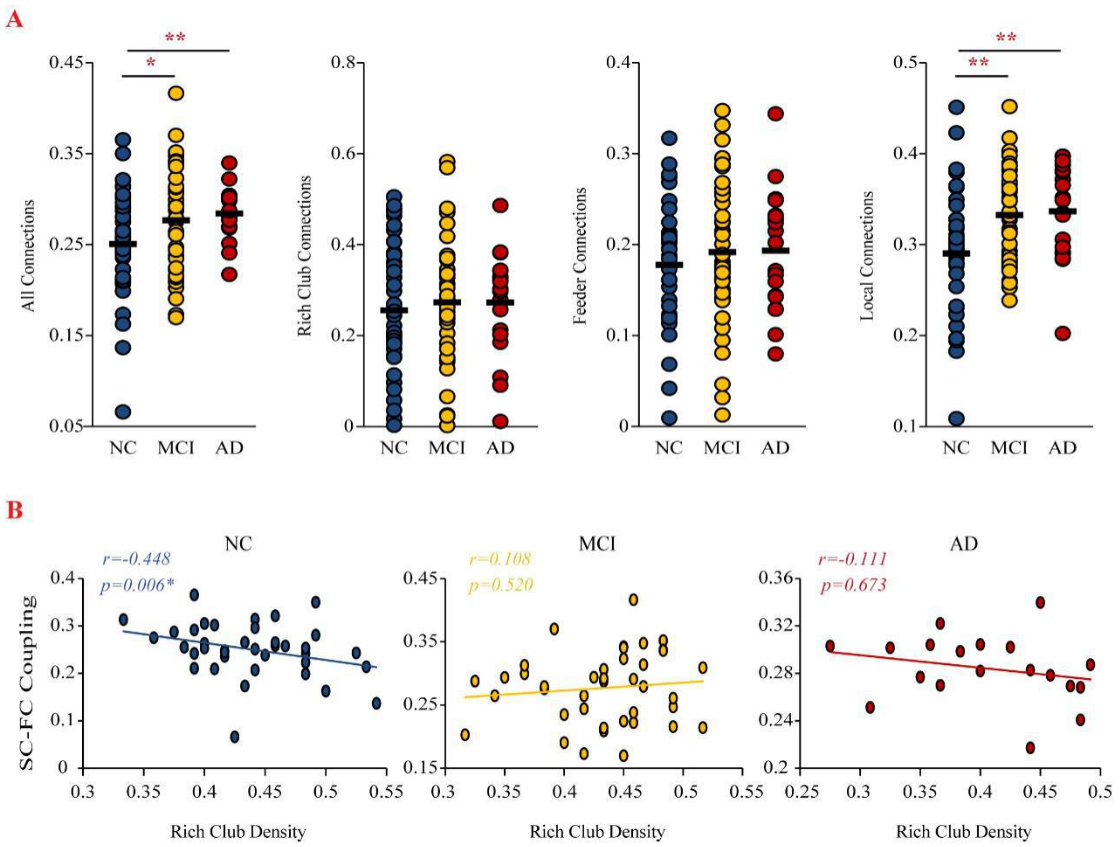

Figure S3. SC-FC Coupling. (A) MCI and AD patients had significantly increased SC-FC coupling compared with the NC group. (B) The correlations between the SC-FC coupling and the rich-club density.

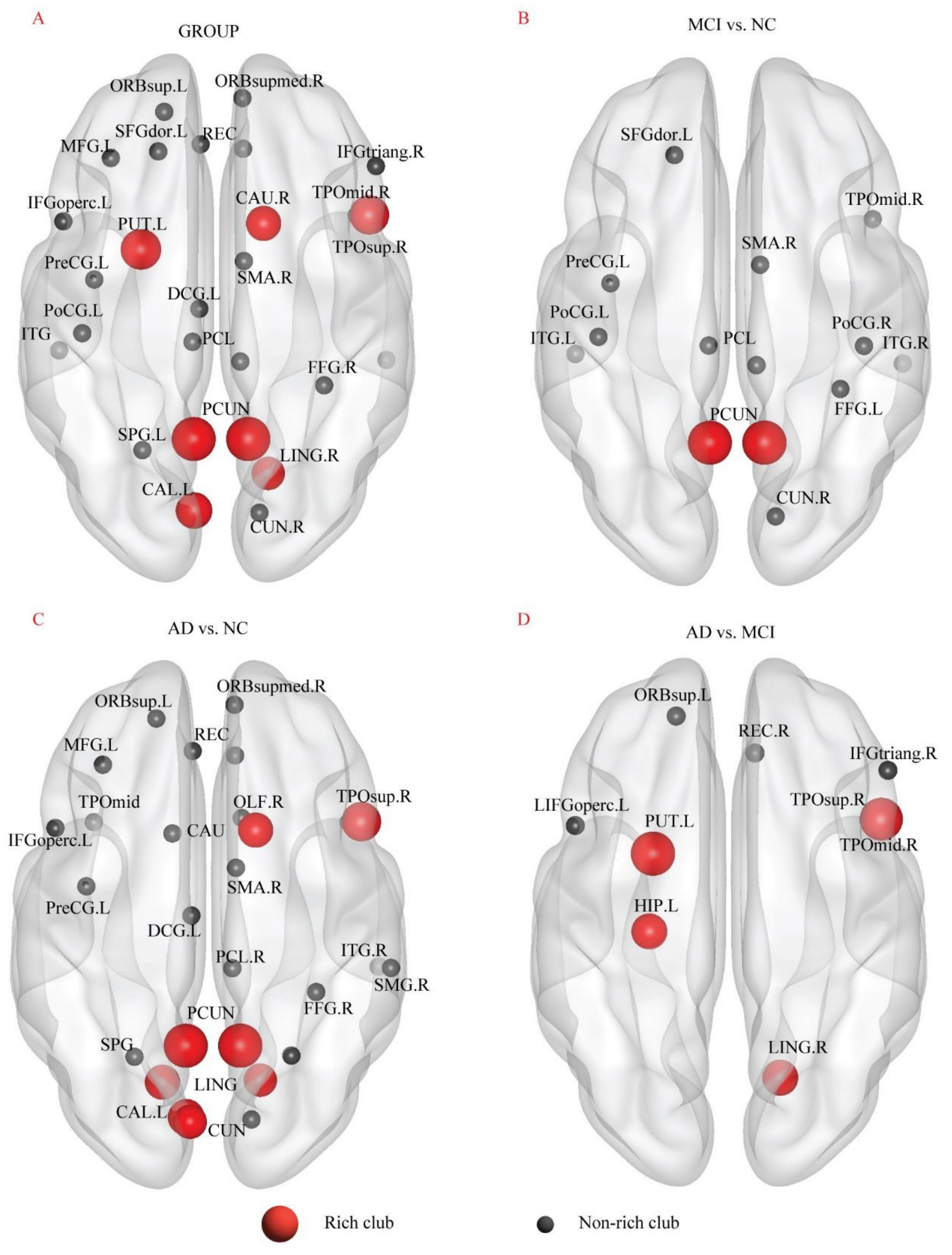

Figure S4. Alzheimer’s disease-related alterations in regional efficiency and structural connectivity.

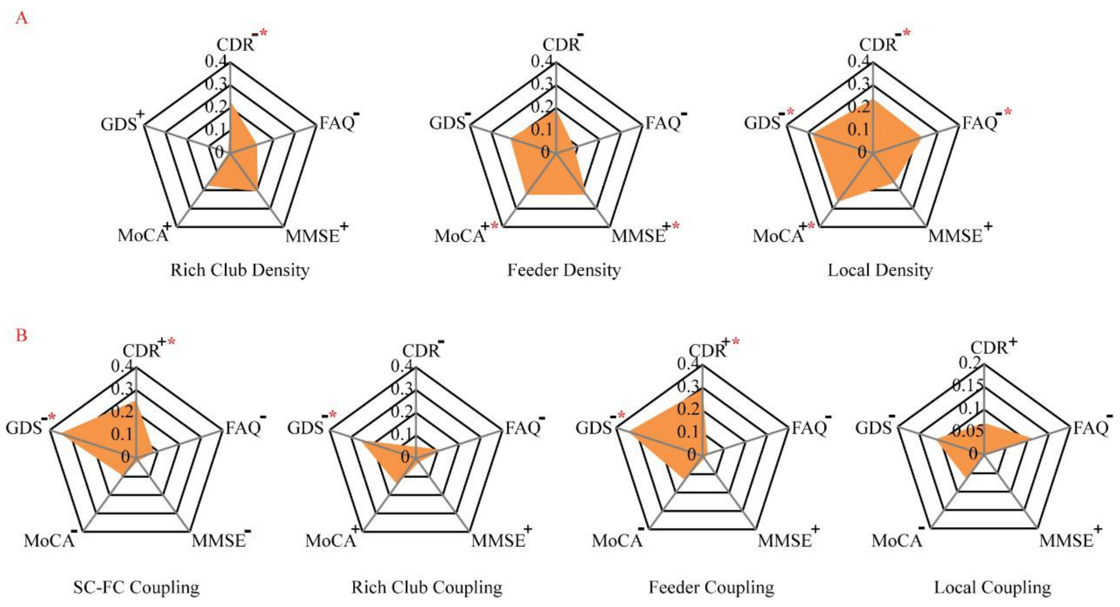

Figure S5. The relationship between connection density and coupling metrics and clinical performance.
